# Genomic prediction and association analyses for breeding parthenocarpic blueberries

**DOI:** 10.1093/hr/uhaf086

**Published:** 2025-03-21

**Authors:** Juliana Cromie, Ryan P Cullen, Camila Ferreira Azevedo, Luis Felipe V Ferrão, Felix Enciso-Rodriguez, Juliana Benevenuto, Patricio R Muñoz

**Affiliations:** Blueberry Breeding and Genomics Laboratory, Horticultural Sciences Department, University of Florida, 2560 Hull Rd, Gainesville, FL 32611, USA; Blueberry Breeding and Genomics Laboratory, Horticultural Sciences Department, University of Florida, 2560 Hull Rd, Gainesville, FL 32611, USA; Blueberry Breeding and Genomics Laboratory, Horticultural Sciences Department, University of Florida, 2560 Hull Rd, Gainesville, FL 32611, USA; Statistics Department, Federal University of Viçosa, Av. Peter Henry Rolfs, s/n, Viçosa, MG 36570-900, Brazil; Blueberry Breeding and Genomics Laboratory, Horticultural Sciences Department, University of Florida, 2560 Hull Rd, Gainesville, FL 32611, USA; Blueberry Breeding and Genomics Laboratory, Horticultural Sciences Department, University of Florida, 2560 Hull Rd, Gainesville, FL 32611, USA; Blueberry Breeding and Genomics Laboratory, Horticultural Sciences Department, University of Florida, 2560 Hull Rd, Gainesville, FL 32611, USA; Blueberry Breeding and Genomics Laboratory, Horticultural Sciences Department, University of Florida, 2560 Hull Rd, Gainesville, FL 32611, USA

## Abstract

Parthenocarpy is a desirable trait that enables fruit set in the absence of fertilization. While blueberries typically depend on pollination for optimal yield, certain genotypes can produce seedless fruits through facultative parthenocarpy, eliminating the need for pollination. However, the development of parthenocarpic cultivars has remained limited by the challenge of evaluating large breeding populations. Thus, establishing molecular breeding tools can greatly accelerate genetic gain for this trait. In the present study, we evaluated two blueberry breeding populations for parthenocarpic fruit set and performed genome-wide association studies (GWAS) to identify markers and candidate genes associated with parthenocarpy. We also compared the predictive ability (PA) of three molecular breeding approaches, including (i) genomic selection (GS); (ii) GS *de novo* GWAS (GS*dn*GWAS), which incorporates significant GWAS markers into the GS model as prior information; and (iii) *in silico* marker-assisted selection (MAS), where markers from GWAS were fitted as fixed effects with no additional marker information. GWAS analyses identified 55 marker–trait associations, revealing candidate genes related to phytohormones, cell cycle regulation, and seed development. Predictive analysis showed that GS*dn*GWAS consistently outperformed GS and MAS, with PAs ranging from 0.21 to 0.36 depending on the population of study and the specific markers utilized. MAS showed PAs comparable to GS in some cases, suggesting it could be a cost-effective alternative to genome-wide sequencing. Together, these findings demonstrate that molecular breeding techniques can be used to improve facultative parthenocarpy, offering new avenues to develop high-yielding blueberry varieties that are less reliant on pollination.

## Introduction

As the cultivation of pollinator-dependent crops continues to outpace the availability of managed pollinators, concerns regarding the strain on pollination services and its potential impact on agricultural productivity have heightened [1–3]. This issue is further compounded by climate change, declines in pollinator health, and the loss of habitat for native pollinators [4–6]. Parthenocarpy, which enables fruit development in the absence of pollination and fertilization, has drawn attention from plant breeders for its potential to ensure yield in pollinator-limited or stressful abiotic conditions [7–10].

Blueberry (*Vaccinium* spp.) is a globally important berry crop [11], which typically relies on cross-pollination to achieve optimal yield and fruit quality [12–15]. Despite the regular use of managed pollinators, the crop frequently suffers from pollen limitation and subsequent yield loss [16–20]. Various factors contribute to pollination deficits, including adverse weather during the brief bloom period [21], suboptimal pollination by honey bees compared to native ‘buzz’ pollinators [22–24], and uneven visitation to cultivars based on pollinators’ floral preferences [25, 26]. Thus, breeding parthenocarpic blueberry varieties can help to supplement yields in cases of pollination deficit [27–29].

Parthenocarpy has been utilized in several crops to obtain seedless fruits [7, 30]. There are three primary classes of parthenocarpy, including stenospermocarpy, which relies on fertilization and subsequent seed abortion, obligate parthenocarpy, in which all fruits are seedless due to sterility or parthenogenesis, and facultative parthenocarpy, where plants can produce either seedless or seeded berries depending on the incidence of pollination [10, 31]. Blueberry exhibits natural facultative parthenocarpy, which allows for parthenocarpic genotypes to be both selected and used as parents in a breeding program [32, 33]. While pollination generally improves blueberry fruit quality, certain genotypes can yield seedless parthenocarpic fruits of comparable size and quality to those that are open-pollinated [34, 35]. However, the development of parthenocarpic cultivars is limited by the challenge of evaluating large breeding populations, which involves the prevention of pollination through flower emasculation and the exclusion of pollinators. Thus, the establishment a molecular breeding pipeline for this trait can help to minimize the need for intensive phenotyping efforts and expedite genetic gains.

Molecular breeding tools have been used to advanced parthenocarpy in various crops [36–39]. In tomato, more than 20 sources of parthenocarpy have been identified, with few instances of facultative parthenocarpy [10, 40, 41]. Candidate genes and variants linked to phytohormones like auxin, gibberellic acid, and cytokinin have been uncovered through quantitative trait loci (QTL) mapping and genome-wide association studies (GWAS) in tomato, eggplant, and cucumber [42–46]. While marker-assisted selection (MAS) has been implemented to improve parthenocarpy in some cases [36, 47], numerous studies indicate a complex genetic architecture with several important QTL involved [46, 48, 49]. Despite this, comprehensive strategies tailored for quantitative traits, such as genomic selection, have yet to be applied for facultative parthenocarpy to our knowledge.

As reported in other fruit species, we hypothesized that parthenocarpy is highly genetically controlled in blueberries and therefore molecular tools could be designed to support breeding decisions. To this end, we evaluated the diversity and potential for facultative parthenocarpy in two southern highbush blueberry breeding populations comprising a total of 505 genotypes. To aid in the development of molecular breeding tools, we identified markers linked to parthenocarpy through GWAS analyses and investigated the potential candidate genes within their surrounding genomic regions. Furthermore, we compared the predictive ability (PA) of three molecular breeding strategies, including genomic selection (GS), GS *de novo* GWAS (GS*dn*GWAS), and *in silico* marker-assisted selection (MAS), considering an interpopulation cross-validation framework to simulate implementation within a real breeding program. Together, we present the largest genomic study discussing new tools for addressing pollination issues in the *Vaccinum* genus.

## Results

### Parthenocarpy phenotype

**Figure 1:**
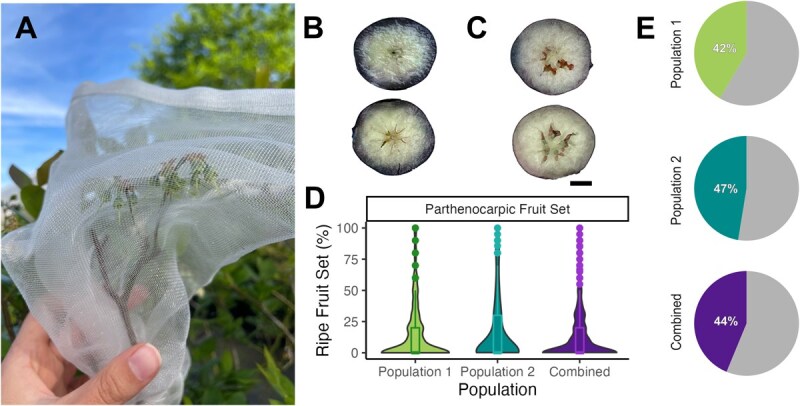
**Assessment of parthenocarpy in two southern highbush blueberry breeding populations.** (A) The parthenocarpy treatment included emasculation of flowers and exclusion of pollinators for 10 flowers of each genotype to prevent any possibility of pollination or fertilization. (B) Facultative parthenocarpic genotypes yielded seedless fruits after emasculation treatment. (C) The same genotype yielded seeded fruits from open pollination in untreated flowers. (D) The raw phenotypic distribution of parthenocarpy for independent and combined populations is presented in a violin plot. (E) Pie charts illustrate the proportion of individuals in each population displaying parthenocarpic fruit set of ≥10%.

A diverse collection of 505 southern highbush blueberry genotypes, comprising two breeding populations, were assessed for parthenocarpic fruit set (Fig. 1A–D). Phenotypic values ranged from 0% to 100% out of 10 unpollinated flowers, with 44% of the genotypes exhibiting at least 10% parthenocarpic fruit set (Fig. 1E). The mean parthenocarpic fruit set for each population was 15% and 17%, respectively. Narrow-sense heritability for parthenocarpy considering both populations was calculated as 0.22.

### Genome-wide association studies for parthenocarpy

**Figure 2:**
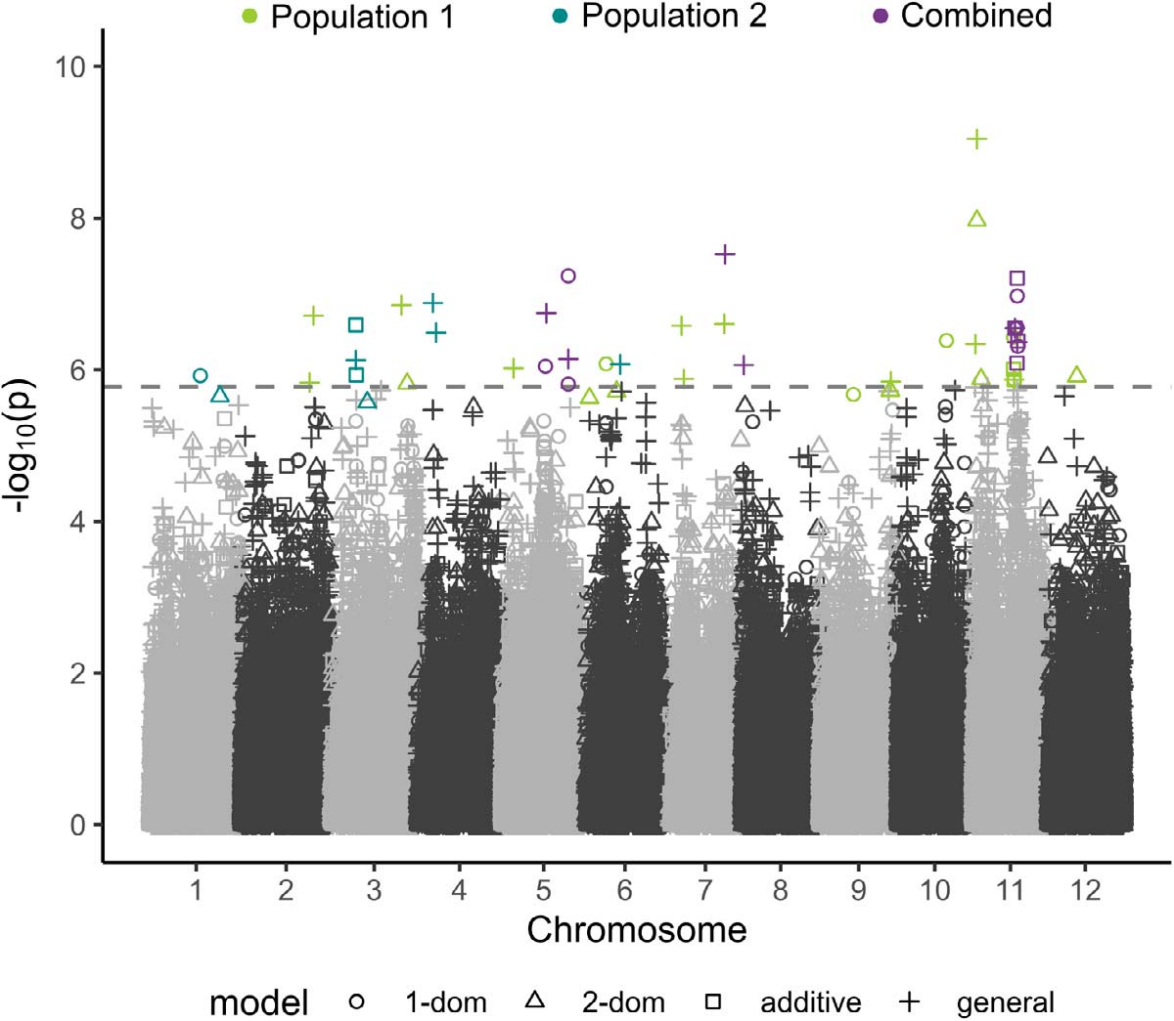
**GWAS results for parthenocarpic fruit set.** Manhattan plots show marker–trait associations for each population (color) and tetraploid gene action model (shape). The most stringent significance threshold for all GWAS analyses is indicated by the dashed horizontal line, and was calculated using the ‘M.eff’ multiple-testing correction (α = 0.05) considering an additive gene action model.

A total of 59 912 SNPs across 12 chromosome-scale scaffolds were tested for association with parthenocarpy (Figs S1–S3). Manhattan plots displaying the results of the GWAS are presented for each population separately and combined (Fig. 2). Independent analysis of the first population (POP1) revealed 29 significant marker–trait associations on chromosomes 2, 3, 5, 6, 7, 8, 9, 10, 11, and 12. The second population (POP2) exhibited nine associations on chromosomes 1, 3, 4, and 6. In the combined analysis (POP1 and POP2), 17 significant SNPs were detected on chromosomes 5, 7, 8, and 11. In all cases, individual markers explained <15% of the phenotypic variation for parthenocarpic fruit set (Table 1).

**Table 1:** All significant SNPs associated with parthenocarpy identified from GWAS analyses.

**Population**	**Chr**	**Position**	**Ref/Alt**	**Model**	**Thresh.**	**Score**	**Effect**	***R*** ^**2**^	***P*-val**	**MAF**
Population 1	2	34110377	C/T	General	5.78	5.83	NA	0.01	0.199	0.03
Population 1	2	36035768	C/T	General	5.78	6.71	NA	0.08	0.000	0.07
Population 1	3	34991045	C/T	General	5.78	6.85	NA	0.09	0.000	0.21
Population 1	3	37811043	C/T	2-dom-ref	5.6	5.82	−0.31	0.00	0.871	0.09
Population 1	5	5776698	C/G	General	5.78	6.02	NA	0.04	0.012	0.08
Population 1	6	2060864	A/T	2-dom-alt	5.42	5.63	−0.34	0.03	0.005	0.20
Population 1	6	10348201	A/G	1-dom-ref	5.73	6.08	0.17	0.06	0.000	0.34
Population 1	6	15569023	A/G	2-dom-ref	5.6	5.71	−0.32	0.00	0.465	0.06
Population 1	7	8421613	G/A	General	5.78	6.58	NA	0.05	0.004	0.17
Population 1	7	9545305	TC/TT	General	5.78	5.88	NA	0.00	0.935	0.01
Population 1	7	29690597	G/A	General	5.78	6.61	NA	0.03	0.047	0.19
Population 1	8	1744735	G/C	2-dom-alt	5.42	5.52	−0.27	0.01	0.221	0.20
Population 1	8	5542221	C/T	1-dom-alt	5.22	5.31	−0.37	0.03	0.002	0.23
Population 1	9	16819984	A/G	1-dom-alt	5.22	5.68	−0.34	0.02	0.013	0.18
Population 1	9	35167278	G/A	2-dom-ref	5.6	5.72	−0.26	0.01	0.219	0.08
Population 1	9	35452313	C/T	General	5.78	5.84	NA	0.02	0.219	0.10
Population 1	9	36318057	G/A	1-dom-alt	5.22	5.47	−0.15	0.01	0.156	0.42
Population 1	10	24262910	C/A	1-dom-alt	5.22	6.39	−0.31	0.04	0.001	0.49
Population 1	11	1833631	G/C	General	5.78	6.34	NA	0.02	0.037	0.04
Population 1	11	2581098	G/A	General	5.78	9.04	NA	0.01	0.641	0.12
Population 1	11	2581098	G/A	2-dom-ref	5.6	7.97	−0.29	0.00	1.000	0.12
Population 1	11	4611151	T/C	2-dom-ref	5.6	5.88	−0.35	0.00	0.790	0.06
Population 1	11	5603261	C/G	2-dom-alt	5.42	5.53	0.28	0.00	0.429	0.07
Population 1	11	20780807	A/C	Additive	5.78	6.01	−0.17	0.01	0.169	0.02
Population 1	11	20780807	A/C	1-dom-ref	5.73	6.43	−0.19	0.00	0.343	0.02
Population 1	11	21099188	T/C	1-dom-ref	5.73	5.87	−0.18	0.00	1.000	0.02
Population 1	11	21099188	T/C	Additive	5.78	5.87	−0.18	0.00	1.000	0.02
Population 1	11	21099188	T/C	General	5.78	5.87	NA	0.00	1.000	0.02
Population 1	12	14473779	C/T	2-dom-ref	5.6	5.91	0.12	0.02	0.016	0.46
Population 2	1	24763902	G/A	1-dom-alt	5.17	5.92	−0.51	0.15	0.000	0.32
Population 2	1	34549718	C/T	2-dom-ref	5.56	5.65	−0.44	0.12	0.000	0.12
Population 2	3	12605530	A/T	General	5.76	6.13	NA	0.09	0.000	0.02
Population 2	3	12605530	A/T	Additive	5.76	6.59	−0.36	0.00	1.000	0.02
Population 2	3	12832095	A/G	Additive	5.76	5.93	−0.33	0.00	0.601	0.02
Population 2	3	18284138	C/T	2-dom-ref	5.56	5.57	−0.41	0.07	0.000	0.09
Population 2	4	8103119	T/C	General	5.76	6.88	NA	0.00	0.804	0.08
Population 2	4	9662988	T/A	General	5.76	6.49	NA	0.10	0.000	0.08
Population 2	6	17667007	C/T	General	5.76	6.07	NA	0.06	0.003	0.06
Combined	5	21298294	A/G	1-dom-ref	5.74	6.05	0.11	0.02	0.002	0.10
Combined	5	21719536	C/T	General	5.78	6.75	NA	0.06	0.000	0.06
Combined	5	32652631	AT/AG	1-dom-ref	5.74	5.81	0.35	0.01	0.065	0.32
Combined	5	32652671	C/T	General	5.78	6.14	NA	0.00	0.873	0.34
Combined	5	32652671	C/T	1-dom-ref	5.74	7.24	0.41	0.00	1.000	0.34
Combined	7	29690597	G/A	General	5.78	7.52	NA	0.06	0.000	0.19
Combined	8	723946	A/G	General	5. 78	6.06	NA	0.02	0.009	0.14
Combined	11	21099188	T/C	1-dom-ref	5.74	6.55	−0.19	0.00	1.000	0.02
Combined	11	21099188	T/C	Additive	5.78	6.55	−0.19	0.00	1.000	0.02
Combined	11	21099188	T/C	General	5.78	6.55	NA	0.00	1.000	0.02
Combined	11	22139008	G/A	Additive	5. 78	6.09	−0.17	0.00	0.270	0.02
Combined	11	22139008	G/A	1-dom-ref	5.74	6.56	−0.20	0.00	0.410	0. 02
Combined	11	22248486	T/A	1-dom-ref	5.74	6.97	−0.21	0.00	1.000	0.02
Combined	11	22248486	T/A	Additive	5.78	7.21	−0.20	0.00	1.000	0.02
Combined	11	22248486	T/A	General	5.78	6.36	NA	0.00	1.000	0.02
Combined	11	22561347	A/G	Additive	5. 78	6.49	−0.19	0.00	0.735	0.02
Combined	11	22561347	A/G	1-dom-ref	5.74	6.31	−0.19	0.000	0.828	0.02

Thresh is the significance threshold for a given gene action model within each population; score indicates the -log_10_(p) value; effect is the coefficient of the marker; *R*^2^ is the proportion of phenotypic variance explained by a multiple QTL model using backward elimination; *P*-val is the *P*-value attributed to each *R*^2^ value; MAF is minor allele frequency in the population.

Altogether, 55 marker–trait associations were detected using tetraploid genetic parameterizations, including additive (8), simplex dominance (15), duplex dominance (11), and general (21) models. Notably, the majority of significant marker–trait associations were found to be population-specific, with many exhibiting different allele frequencies in each independent population (Fig. S6). However, significant marker–trait associations were identified on chromosome 6 in both breeding populations, with markers positioned proximally within a 2 Mb window. Additionally, two loci on chromosomes 7 and 11 were found to be significant in both POP1 and the combined analysis.

### Candidate gene mining

Genomic regions (±100 kb) flanking the significant SNPs identified from GWAS analyses were investigated for candidate genes related to parthenocarpy. Of the 390 genes within these windows, 232 (59%), and 226 (58%) were functionally annotated *in silico* by the PANZZER2 and eggNOG-mapper servers, respectively. Candidate genes related to phytohormone regulation, cell division, embryogenesis, and seed development were found in these regions. A selected list of top candidate genes with literature support is presented in Table 2. The potential roles of these genes in parthenocarpy are further presented in the discussion section.

**Table 2:** A list of selected candidate genes related to parthenocarpy.

**Population**	**Gene name**	**Chr**	**Start**	**End**	**Description**	**PFAMs**	**References**
Population 2	VaccDscaff1-snap-gene-247.18	1	24700091	24702355	Peroxidase	Peroxidase	[68], [69], [70], [71]
Combined	VaccDscaff7-augustus-gene-327.28	5	32695014	32697554	Autophagy-related 2	-	[72], [73]
Population 2	VaccDscaff11-augustus-gene-177.31	6	17668793	17677635	Calmodulin-binding protein 60 B-like	Calmodulin_bind	[74], [75], [76]
Population 1	VaccDscaff12-processed-gene-83.4	7	8357513	8361527	Cytochrome P450	p450	[77], [78], [79], [80]
Population 1	VaccDscaff12-augustus-gene-296.27	7	29618208	29620941	Heavy metal-associated isoprenylated plant protein 39	HMA	[81]
Combined & Population 1	VaccDscaff12-snap-gene-296.36	7	29637070	29639862	WRKY domain-containing protein	WRKY	[82], [83, 84], [85]
Combined	VaccDscaff12-augustus-gene-296.30	7	29651276	29654098	Desiccation protectant protein Lea14 homolog	LEA_2	[74], [86]
Combined	VaccDscaff12-processed-gene-297.0	7	29676797	29681425	S-adenosyl-L-methionine-dependent methyltransferases	Methyltransf_29; zfNARING_2	[87], [88]
Combined	VaccDscaff13-augustus-gene-7.21	8	699713	701850	Ethylene-responsive transcription factor	AP2	[74], [89], [88]
Population 1	VaccDscaff13-snap-gene-17.60	8	1775028	1781304	Polygalacturonase-like protein	Glyco_hydro_28	[90]
Population 1	VaccDscaff17-snap-gene-167.20	9	16733085	16735477	Knotted1-like homeodomain protein liguleless4b (Fragment)	ELK,Homeobox_KN	[91], [92], [93]
Population 1	VaccDscaff17-augustus-gene-355.30	9	35494747	35499415	Auxin-responsive protein	AUX_IAA	[94], [95], [96]
Population 1	VaccDscaff21-augustus-gene-207.21	11	20744582	20753840	Glucomannan 4-beta-mannosyltransferase 9	Glyco_trans_2_3	[97]
Population 1	VaccDscaff21-snap-gene-208.21	11	20830135	20845455	Transcriptional activator DEMETER	PermNACXXC,RRM_DME	[98], [99]

Gene descriptions and annotations are provided from the PANZZER2 software. Protein families from the Pfam database are also indicated. Literature sources are provided for studies that identified these genes as candidates for parthenocarpy.

Among the most noteworthy candidate genes, we identified an ‘auxin-responsive protein’ (*VaccDscaff17-augustus-gene-355.30)* ~42 kb downstream of marker 17_35452313 on chromosome 9 (Table 2). The encoded protein contains an Aux/IAA domain (IPR033389) and shares 47.12% sequence identity with *Arabidopsis thaliana* IAA27 (AT4G29080), an auxin-related gene involved in fertility and fruit enlargement (Fig. S5) [100]. Moreover, we observed a substantial shared amino acid sequence identity (61.34%) between VaccDscaff17-augustus-gene-355.30 and a previously characterized *VcIAA27* (VaccDscaff30-augustus-gene-75.28). Shared conserved domains were also observed between these proteins, suggesting they could possess similar functions. Individuals containing a single copy of the alternate allele of this marker exhibited a mean parthenocarpic fruit set of 45%, while duplex, triplex, and quadruplex genotypes had an average of 13% (Fig. S6).

On the same chromosome, we also discovered a ‘*knotted1-like homeodomain protein*’ (*VaccDscaff17-snap-gene-167.20*) located ~85 kb downstream of the significant marker 17_16819984. Individuals that were homozygous for the reference allele had an average fruit set of 32%, more than twice that of all other genotypes.

In both populations, we identified significant marker–trait associations located within 2 Mb of each other on chromosome 6. In this region, we identified a marker 11_17667007, located 1786 bp upstream of a ‘calmodulin 60B-like protein’, named ‘VaccDscaff11-augustus-gene-177.31’. Genotypes duplex for the alternative allele exhibited a mean parthenocarpic fruit set of 50%, while other allelic dosages had a mean fruit set of 13%.

### Evaluation of predictive breeding methods

**Figure 3:**
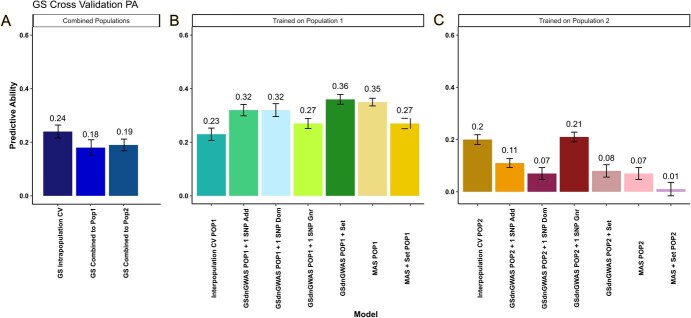
**PA of molecular breeding strategies.** A bar plot showing PA of different breeding and cross-validation schemes. (A) Cross-validation of the GS model trained on the combined population and tested both within the combined population and across each independent population. (B) Interpopulation cross-validation GS, GS*dn*GWAS, and MAS by training on POP1 and predicting POP2. (C) Interpopulation cross-validation by training on POP2 and predicting POP1. GS includes the full marker panel of ~59 k SNPs, GS*dn*GWAS includes the top-performing additive (Add), dominance (Dom), or general (Gnr) markers from GWAS analyses. GS*dn*GWAS Set includes the best set of 2–5 markers from any genetic parameterization with the highest PA. MAS includes a single marker as a fixed effect, with no other marker information. MAS set is the PA of the top-performing marker set identified from GS*dn*GWAS tested within a MAS framework.

To compare the efficacy of genome-wide and targeted molecular breeding approaches, we evaluated the PA of three models: GS, GS*dn*GWAS, and *in silico* MAS (Fig. 3). Overall, genomic prediction showed moderate accuracy, achieving a PA of 0.24 when trained and tested on the combined populations. However, performance declined slightly when the model was tested on the independent populations. In both interpopulation cross-validation schemes, GS*dn*GWAS outperformed GS methodology, although overall predictive abilities were moderate. In the first training–testing scenario, the inclusion of multiple markers as fixed effects resulted in the greatest improvement in PA, with four markers (21_21099188, 12_29690597, 2_34110377, and 2_36035768) exhibiting a PA of 0.36 ± 0.10 (+57%) compared to GS. In the reciprocal training–testing scenario, GS*dn*GWAS modestly improved PA to 0.21 (+5%) when using a single marker exhibiting a general gene action. However, including multiple markers as fixed effects decreased PA to 0.08 ± (−60%). MAS of both a single marker and a set of multiple markers resulted in higher predictive abilities than GS in the first training–testing scenario, with predictive abilities of 0.35 ± 0.08 (+52%) and 0.27 ± 0.11 (+17%), respectively. However, improvements in the reciprocal direction were not observed. Still, the PA obtained using a single general marker exhibited a PA of 0.07 ± 0.12, almost half that obtained by GS using the full marker set.

## Discussion

Parthenocarpy is an important objective for pollination-dependent plant breeders as it reduces reliance on pollination services while securing yields in adverse weather conditions or pollinator-limited environments, such as controlled agriculture systems. Additionally, the facultative parthenocarpy exhibited by blueberry enables elite parthenocarpic genotypes to be both selected and used as parents in the breeding program due to the development of viable seeds upon cross-pollination. In the present study, we observed considerable variability and moderate heritability for natural parthenocarpy in a diverse southern highbush blueberry breeding program, underscoring the potential for integration of this trait into selection criteria. Moreover, through GWAS, we identified significant loci contributing to parthenocarpy, along with the candidate genes within the associated genomic regions. Finally, we compared three common molecular breeding approaches to enhance our understanding of their applicability within a breeding program.

Numerous studies have explored the genetic basis of parthenocarpy, revealing various mechanisms that range from simple to complex. Ehlendeldt and Vorsa (2007) proposed a simple genetic model for northern highbush blueberry, suggesting that parthenocarpy is controlled by a single recessive gene with incomplete dominance [101]. However, F_2_ segregation ratios showed poor fit to a tetrasomic inheritance model. Similar genetic models for parthenocarpy have been reported in cucumber, oil palm, eggplant, and tomato, with mapping studies revealing a small number of regulatory genes exhibiting nonadditive actions [36, 43, 45, 102, 103]. However, some studies indicate that parthenocarpy is instead a quantitative trait, where multiple loci contribute a minor fraction of the phenotypic variance [43, 48, 49]. Our experiment uncovered multiple population-specific marker–trait associations distributed throughout the genome, each accounting for <15% of the variability for parthenocarpic fruit set. Upon further investigation, we found that many of these markers exhibited differences in allele frequencies within each breeding population, limiting our ability to detect the same marker–trait associations across populations using a GWAS analysis. Despite this, subsequent cross-validation of molecular breeding models showed that these markers still enhanced the prediction of parthenocarpy across populations within the context of GS*dn*GWAS, although the extent of improvement depended on the training–testing populations. This finding further supports that the markers identified in GWAS analyses maintain some biological importance for the regulation of parthenocarpy, even though they explained only a small portion of phenotypic variance in each population.

Interesting candidate genes related to phytohormones, seed development, embryogenesis, and cell cycle regulation were also discovered from GWAS analyses (Table 2). We identified *VaccDscaff17-augustus-gene-355.30*, which belongs to the Aux/Indole-3-Acetic Acid (Aux/IAA) family, known for its pivotal role in auxin signaling during fruit development [95, 104]. Furthermore, mutants of AUX/IAA genes have been shown to induce parthenocarpy [94, 105, 106]. Phylogenetic analysis of Arabidopsis AUX/IAA proteins revealed that the blueberry candidate gene *VaccDscaff17-augustus-gene-355.30* clustered closely with auxin-responsive protein IAA27 (AtIAA27) and VaccDscaff30-augustus-gene-75.28, a functionally validated *VcIAA27*. Importantly, these genes exhibit substantial similarity within the conserved domains essential for the functionality of AUX/IAA proteins. Recent studies on *VcIAA27* demonstrated that heterologous overexpression in Arabidopsis led to the development of seedless siliques [100]. Moreover, in tomato, silencing of *SlIAA27* resulted in higher auxin sensitivity and seedless fruit development [107], providing further evidence for the involvement of this gene in parthenocarpy.

A ‘*Knotted1-like (KNOX) homeodomain gene*’, *VaccDscaff17-snap-gene-167.20*, was also discovered. *KNOX* genes encode a group of transcription factors that negatively regulate gibberellic acid biosynthesis, affecting fruit set through the direct repression of *gibberellin 20-oxidase* (*GA20ox*) [108, 109]. In tomato, *KNOX* genes have been implicated for their involvement in parthenocarpy due to their decreased expression in *parthenocarpic fruit* (*pat*) mutants, which exhibit constitutive expression of *GA20ox* [91, 93]. An investigation of *pat* double mutants found that lines also overexpressing *Tomato knotted 2* (*Tkn2*) showed reduced parthenocarpic ability [92]. This finding emphasizes the role of *KNOX* genes in the ‘GA-overdose’ phenotype attributed to *pat* mutants and contributes to the mechanistic understanding of facultative parthenocarpy.

Additionally, both populations revealed neighboring associations on chromosome 6. This region contained a candidate gene *VaccDscaff11-augustus-gene-177.31*, which encodes a ‘calmodulin-binding 60B-like’ protein. Calmodulin-like proteins (CML) have been shown to mediate plant responses to abiotic and biotic stresses, and auxin signaling [110–112]. Analysis of the ~3-kb promoter region of the cucumber *CsCML25* revealed four MYB-binding sites, one WUS-binding site, and several *cis*-acting elements known to interact with auxins [113]. An experiment involving overexpression of this gene increased the rate of parthenocarpy by 86% [114]. Calmodulin-associated genes have also been associated with tomato parthenocarpy through differential gene expression analysis [76].

In the pursuit of an appropriate molecular breeding strategy, we next evaluated the PA of three marker-assisted approaches within a ridge–regression framework. Each approach varies in the molecular information it utilizes and is tailored to a specific genetic architecture [115]. For example, GS leverages genome-wide marker data and is well suited for complex traits influenced by numerous loci with small effects [116, 117]. On the other hand, GS*dn*GWAS provides the same marker information but incorporates a limited number of known QTL regions as fixed effects. This strategy prevents the shrinkage of these marker estimates and enhances the capture of genetic variance guided by biological priors [118]. Conversely, MAS relies on a single marker, or small set of markers, making it ideal for traits controlled by a few major-effect QTLs [119]. To our knowledge, the present study represents the first application of genomic prediction and the largest phenotypic dataset collected for facultative parthenocarpy among horticultural crops.

Results from GS on the combined populations yielded a baseline PA of 0.24, while interpopulation cross-validation exhibited predictive abilities of 0.23 and 0.20, for each training–testing scenario. These values are comparable to those obtained for various fruit quality traits in tetraploid blueberries [120], and other fruit trees [121, 122]. The inclusion of significant GWAS hits as fixed effects within a GS*dn*GWAS framework enhanced predictions of parthenocarpy in both training–testing scenarios, although the extent of these benefits varied depending on the GWAS hits utilized and the allele frequencies of these markers within the training–testing populations. For example, GS*dn*GWAS resulted in substantially higher predictive abilities when training on POP1 to predict POP2, compared to the reciprocal direction. This finding could be explained by the higher number of significant markers associated with parthenocarpy that were identified in the first population (29 versus 11 markers), and the greater strength of their signal. GS*dn*GWAS has shown similar improvements to the prediction of horticultural traits, such as blueberry volatile organic compounds [67], and of agronomic traits in rice [123] and wheat [124]. However, in these cases, the relative performance of GS*dn*GWAS models also varied depending on the species, population, the trait of interest, and its genetic architecture.

We observed similar population-specific outcomes using *in silico* MAS. When training on POP1 to predict POP2, we identified single markers and sets of markers that could predict parthenocarpic fruit set with greater accuracy than GS. However, when MAS models were fit in the reciprocal training–testing scenario, we observed substantially lower predictive abilities. For several markers identified in GWAS of POP1, we observed lower allele frequencies in POP2, which could have enabled more accurate predictions of this population. Additionally, we suspect that genotype-by-environment interactions could influence the expression of parthenocarpy, affecting our ability to detect common significant marker–trait associations in both populations. Still, one single marker identified in POP2 exhibited a PA of nearly half that of GS, demonstrating the relative importance of these markers in the regulation of parthenocarpy. Therefore, while GS*dn*GWAS provides the most robust prediction of this trait, the development of diagnostic marker assays could also support molecular breeding efforts without significant investments in genome-wide sequencing.

## Conclusions

Through the utilization of various molecular breeding tools, we identified several loci and candidate genes for facultative parthenocarpy and determined the optimal predictive breeding strategy for its improvement. We show that although single markers may be useful to predict parthenocarpy in a MAS approach, optimal predictions were obtained using GS*dn*GWAS. These findings underscore the advantage of leveraging prior biological information from GWAS to improve the prediction of complex traits. Furthermore, we highlight the utility of small-effect QTL identified from GWAS in the prediction of new breeding populations. Overall, this work represents a step forward in our understanding of facultative parthenocarpy, exhibited by blueberry, and offers a robust prediction framework for implementation in modern breeding programs.

## Materials and Methods

### Population and plant materials

In this study, 505 southern highbush blueberry genotypes were analyzed. These genotypes were sourced from two groups of advanced selections developed as part of the clonal selection trials from the University of Florida Blueberry Breeding program planted in Waldo, FL, USA (29.792664, −82.132109). All individuals belong to the same breeding pool and are related by pedigree at different levels. For the validation of genomic analyses, these populations are designated as Population 1 (POP1) and Population 2 (POP2), and they include 311 and 194 individuals, respectively. Phenotypic evaluation of POP1 and POP2 was conducted during the 2021 and 2022 seasons when plants were 7 and 8 years of age, respectively. Additionally, 50 genotypes from POP1 were evaluated in 2022 to connect both datasets and correct for eventual confounding effects of the year and age of the plants.

### Measuring parthenocarpic capacity

For each genotype, a single raceme was bagged with clear plastic mesh netting during the bud stage. Prior to anthesis, 10 flowers were emasculated by peeling back the corolla and removing stamens with a pair of forceps. All untreated flowers and remaining flower buds were removed, and the mesh bag was reinstalled to prevent pollination. Parthenocarpic capacity was measured as the proportion of ripe fruit set out of 10 flowers in response to emasculation and pollinator exclusion. All fruits were checked for seeds to ensure the absence of fertilization.

### Genotypic data

Young leaves were collected from each genotype and submitted for DNA extraction and targeted sequence capture genotyping at RAPiD Genom10372ics (Gainesville, FL, USA), as described by Ferrão et al. [50]. A total of 10 K probes of 120-mer were used for target enrichment. Sequencing was conducted via 150-cycle paired-end runs using the IlluminaHiSeq2000 platform [51]. Trimmed and filtered reads were then aligned to the primary haplotype of the reference genome *Vaccinium corymbosum* cv. ‘Draper’ [52] using Mosaik v.2.2.3 software [53]. Variant calling was performed via FreeBayes v.1.3.2 [54], targeting the probe regions and considering a minimum mapping quality of 10. Single nucleotide polyphormisms (SNPs) were then filtered for biallelic markers only with a maximum of 50% missing data. Tetraploid allelic dosage was assigned based on the proportion of alternative and reference allele counts using the ‘updog’ R package [55]. Missing genotypes were imputed by the mean of each locus. The final SNP set included 59 912 markers that were used for all subsequent genome-wide association and genomic prediction analyses.

### Phenotypic analysis

Best linear unbiased estimators (BLUEs) were used to connect phenotypic information across years, including both populations and considering checks, as follows: $\boldsymbol{y}=\mathbf{1}\mathrm{\mu} +{\boldsymbol{X}}_{\mathbf{1}}{\boldsymbol{g}}_{\mathbf{1}}+{\boldsymbol{X}}_{\mathbf{2}}{\boldsymbol{g}}_{\mathbf{2}}+{\boldsymbol{X}}_{\mathbf{3}}\boldsymbol{\beta} +\boldsymbol{e}$ (1), where $\boldsymbol{y}$ is the vector of phenotypes for individuals collected in POP1 and POP2, $\mathrm{\mu}$ is the population phenotypic mean, and $\mathbf{1}$ is a vector of the same dimension of $\boldsymbol{y}$ being all elements equal to 1. Following a similar model reported by Pastina et al. [56] and De Lara et al. [57], we separated the genotypic effect $\boldsymbol{g}$ into two groups, where ${\boldsymbol{g}}_{\mathbf{1}}$ is a fixed effect vector of checks connecting both years of evaluation with an incidence matrix (${\boldsymbol{X}}_{\mathbf{1}}$), ${\boldsymbol{g}}_{\mathbf{2}}$ is a fixed effect vector of test genotypes, with an incidence matrix of (${\boldsymbol{X}}_{\mathbf{2}}$), and $\boldsymbol{\beta}$ is the fixed effect of the year, with an incidence matrix (${\boldsymbol{X}}_{\mathbf{3}}$). The residual term was assumed as $\boldsymbol{e}\sim MVN\left(\mathbf{0},\boldsymbol{I}{\sigma}_e^2\right)$, where $\boldsymbol{I}$ is the identity matrix and ${\sigma}_e^2$ is the error variance.

To estimate genetic parameters, we fit a similar model where the year and check genotypes were considered as fixed effects, and the genetic effect of the test genotypes was instead considered as a random effect. For the random genetic term, we computed a pedigree-based heritability value by assuming ${\boldsymbol{g}}_{\mathbf{2}}\sim MVN\big(\mathbf{0},\boldsymbol{A}{\mathrm{\sigma}}_g^2\big)$, where $\boldsymbol{A}$ is the additive relationship matrix constructed using the ‘AGHmatrix’ R package [58] following statistical methodology for autotetraploid species from [59], and ${\sigma}_g^2$ is the additive genetic variance. Narrow-sense heritability (h^2^) was computed by ${h}^2=\frac{\sigma_a^2}{\sigma_a^2+{\sigma}_e^2}$ (2). All models were fitted using the ‘ASReml’ R package [60].

### Genome-wide association studies

GWAS were conducted on each population independently and combined. Individual GWAS considered raw phenotypic values, and the combined analysis utilized BLUE values corrected for the year effect, as described in the previous section. For the GWAS analysis, a Q + K model was implemented using the GWASpoly R package [61]. The Q matrix incorporated the first five principal components as covariates to address population structure, and the K covariance matrix was generated using the leave-one-chromosome out (LOCO) method to account for polygenic effects. All tetraploid gene action models were tested, including additive, dominance (simplex, ‘1-dom’, and duplex, ‘2-dom’), and general models. Significance thresholds for marker–trait associations were adjusted using the ‘M.eff’ multiple-testing correction option, considering *α = 0.05* and minimum minor allele frequency (MAF) of 1%*.* This method estimates an effective number of markers using linkage disequilibrium (LD) between markers [62]. Quantile–quantile (Q–Q) plots were generated to verify model fit, and LD decay was visualized using the ‘LD.plot’ function.

### Candidate gene identification

Genomic windows of ±100 kb surrounding each significant marker discovered in the GWAS were mined for candidate genes. Gene homology and functional annotation data were gathered from NCBI BLAST, Gene Ontology (GO), KEGG pathway, Conserved Domain Database (CDD), and protein family database (PFAM) using the ‘PANZZER2’ [63] and eggNOG-mapper [64] software. Genes related to cell cycle, fruit set, fruit development, phytohormone production and signaling, and those previously identified in the literature to be involved in parthenocarpy were included.

To further investigate the structure and function of candidate genes, blueberry gene and protein sequences were obtained from the Genome Database for Vaccinium (https://www.vaccinium.org/), and homologous genes were obtained from NCBI (http://www.ncbi.nlm.nih.gov). Amino acid sequences were aligned using the MEGA 11 software [65].

### Predictive breeding strategies (GS, GS*dn*GWAS, and MAS)

Three molecular breeding strategies were compared for their ability to predict parthenocarpic capacity: GS, GS*dn*GWAS, and *in silico* MAS. As a benchmark, GS was performed on the combined populations using random cross-validation. In all other scenarios, an interpopulation cross-validation scheme was used in which one population was used for model training, and the other for model testing. Additionally, we conducted cross-validation by training on the combined populations and testing on each independent population separately. To account for differences in population size, all models were fit using a random sample of 175 individuals from the training population to predict a random 50 individuals from the testing population. Cross-validation was repeated for 30 iterations.

All prediction models utilized a ridge–regression model to obtain genomic estimated breeding values. The following statistical model was considered: $\boldsymbol{y}=\mathbf{1}\mathrm{\mu} +\boldsymbol{X}\boldsymbol{\beta } +\boldsymbol{Zu}+\boldsymbol{\varepsilon}$ (1) where ***y*** is the vector of phenotypic observations, $\mathrm{\mu}$is the overall mean, $\boldsymbol{\beta}$ is the vector of fixed effects with incidence matrix $\boldsymbol{X}$***,***  $\boldsymbol{u}\sim MVN\left(\mathbf{0},\boldsymbol{I}{\mathrm{\sigma}}_u^2\right)$ is the vector of random marker effects with incidence matrix $\boldsymbol{Z}$, and $\boldsymbol{\varepsilon}$ is the residual vector with $\boldsymbol{\varepsilon} \sim MVN\left(\mathbf{0},\boldsymbol{I}{\mathrm{\sigma}}_{\varepsilon}^2\right)$, where $\boldsymbol{I}$ is the identity matrix, and ${\mathrm{\sigma}}_{\varepsilon}^2$ is the residual variance. All models were implemented using the ‘rrBLUP’ R package [66]. For GS, genome-wide SNP markers were considered as random effects, and no fixed effects were included ($\boldsymbol{\beta} =\mathbf{0}$). For GS*dn*GWAS, up to five SNP markers were included as a fixed effect, with all other markers as random effects. The rationale behind GS*dn*GWAS is to avoid shrinkage of those SNP effects identified as significant in GWAS, providing more flexibility for the estimation of markers with a larger effect. The selection of SNPs with significant effects from GWAS analyses for inclusion as predictors in GS*dn*GWAS models was determined through bidirectional stepwise regression. In this process, markers included in the final model were tested in all possible combinations in sets of two to five markers. In the MAS regression model, each significant marker from GWAS analyses was tested individually and considered as a fixed effect with no additional marker information ($\boldsymbol{u}=\mathbf{0}$). Furthermore, the top-performing marker set from GS*dn*GWAS was also evaluated within a MAS framework. Importantly, to avoid overfitting, all tested markers for GS*dn*GWAS and MAS were derived from GWAS of the independent populations. Similar molecular testing strategies were reported by Ferrão et al. [67]. PAs were computed as the Pearson’s correlation between the predicted and observed phenotypic values.

## Supplementary Material

Web_Material_uhaf086

## Data Availability

The genomic and phenotypic data are available as supplementary material.
