# Supplementary figures and images for: Genomic prediction and association analyses for breeding parthenocarpic blueberries
Source: Hortic Res. 2025 Mar 21;12(7):uhaf086. doi: 10.1093/hr/uhaf086 (PMC12064955; doi:10.1093/hr/uhaf086)

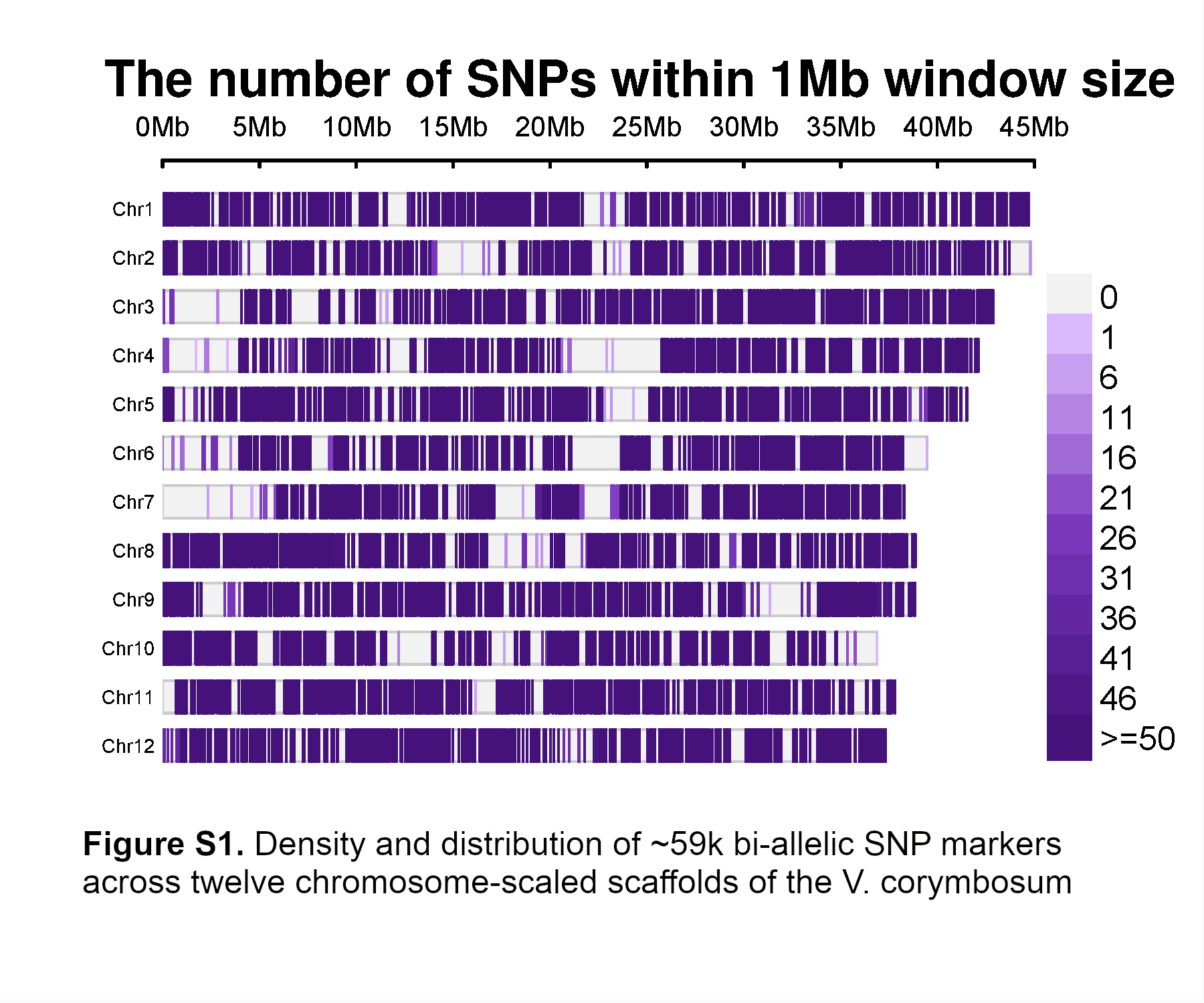

Supplement: Web_Material_uhaf086 [file web_material_uhaf086.zip › S1_Draper_SNPdensity_N.jpg]

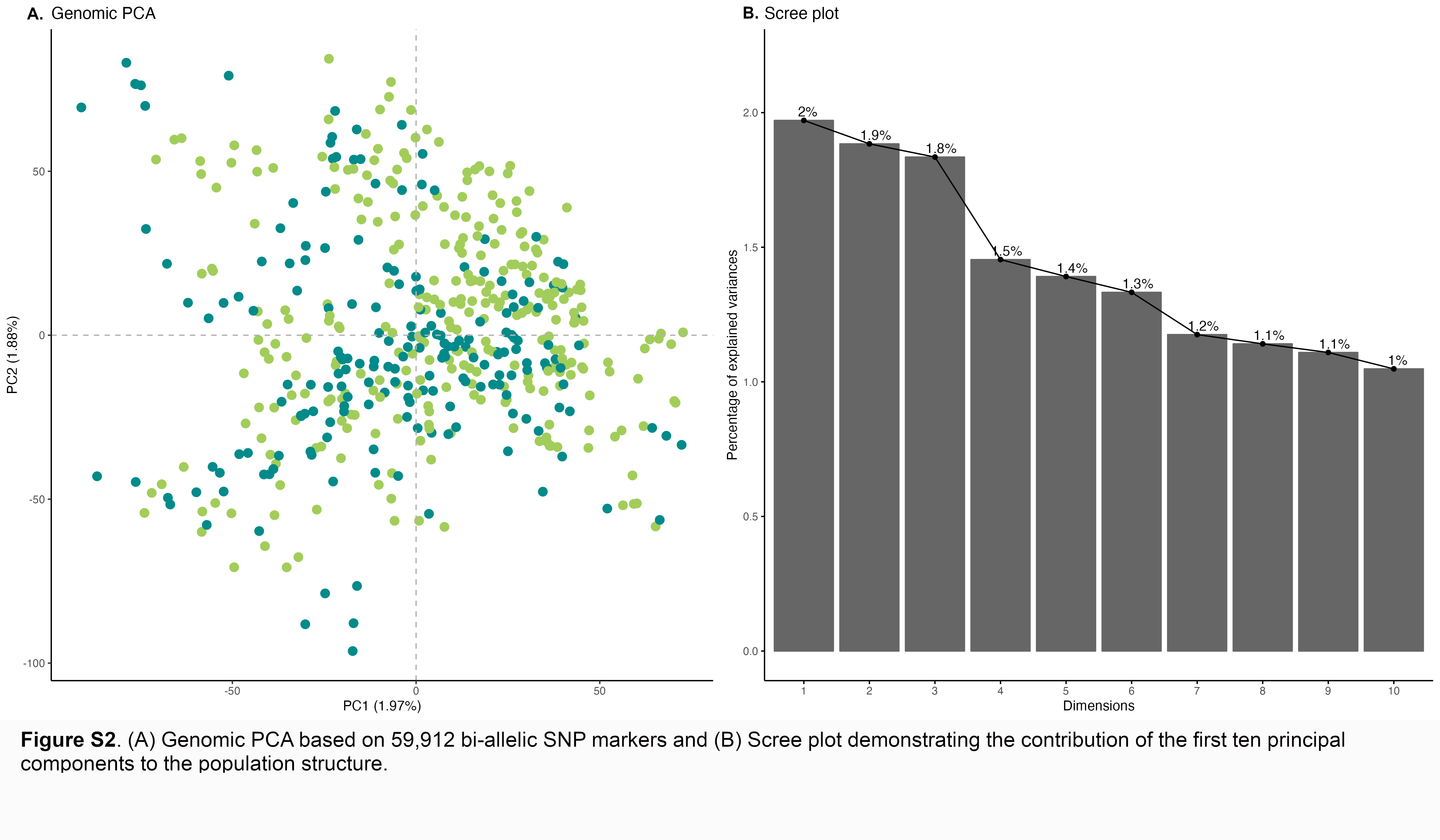

Supplement: Web_Material_uhaf086 [file web_material_uhaf086.zip › S2_GenomicPCA_N.jpg]

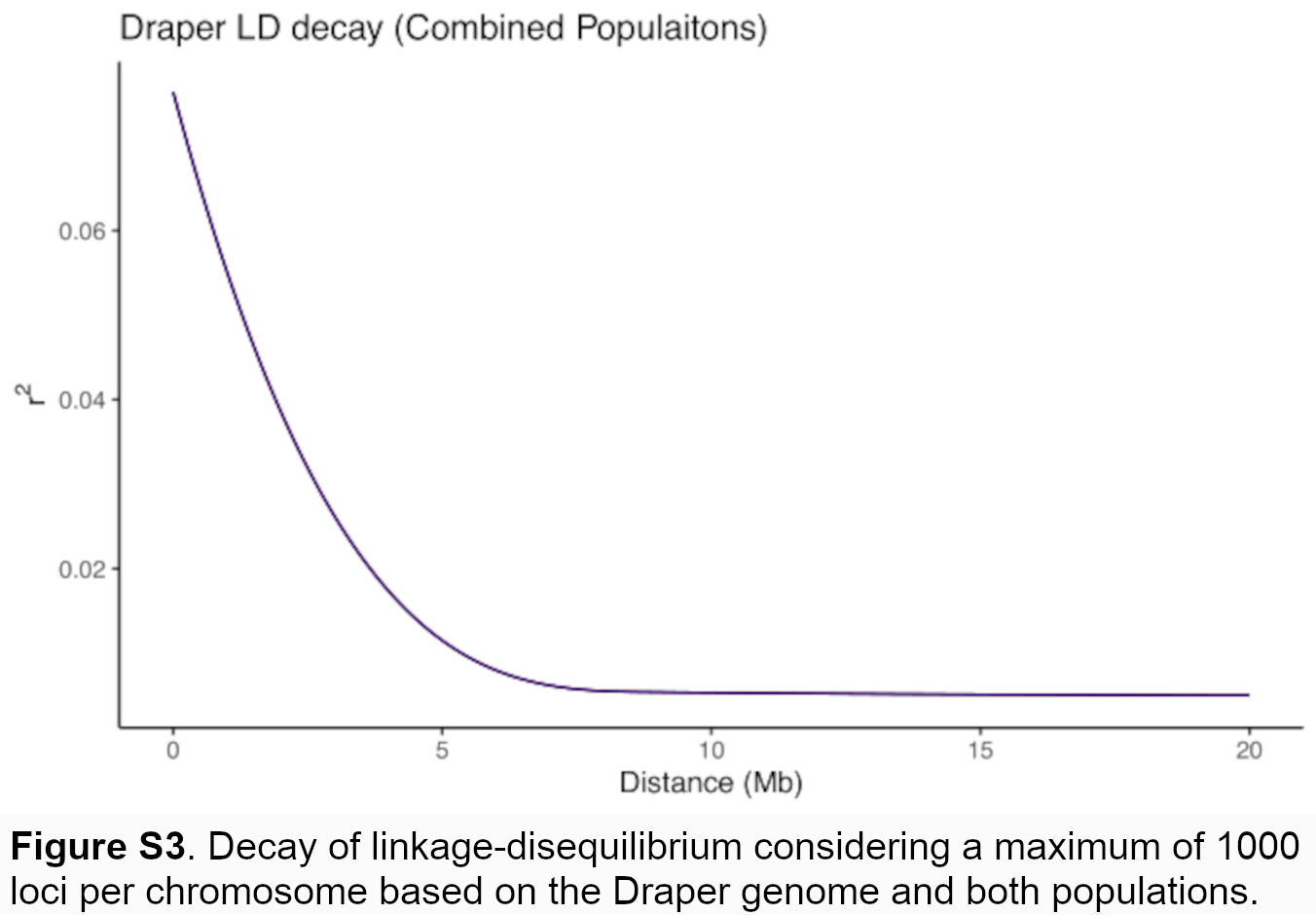

Supplement: Web_Material_uhaf086 [file web_material_uhaf086.zip › S3_Draper_LD_CombinedPopulations_N.jpg]

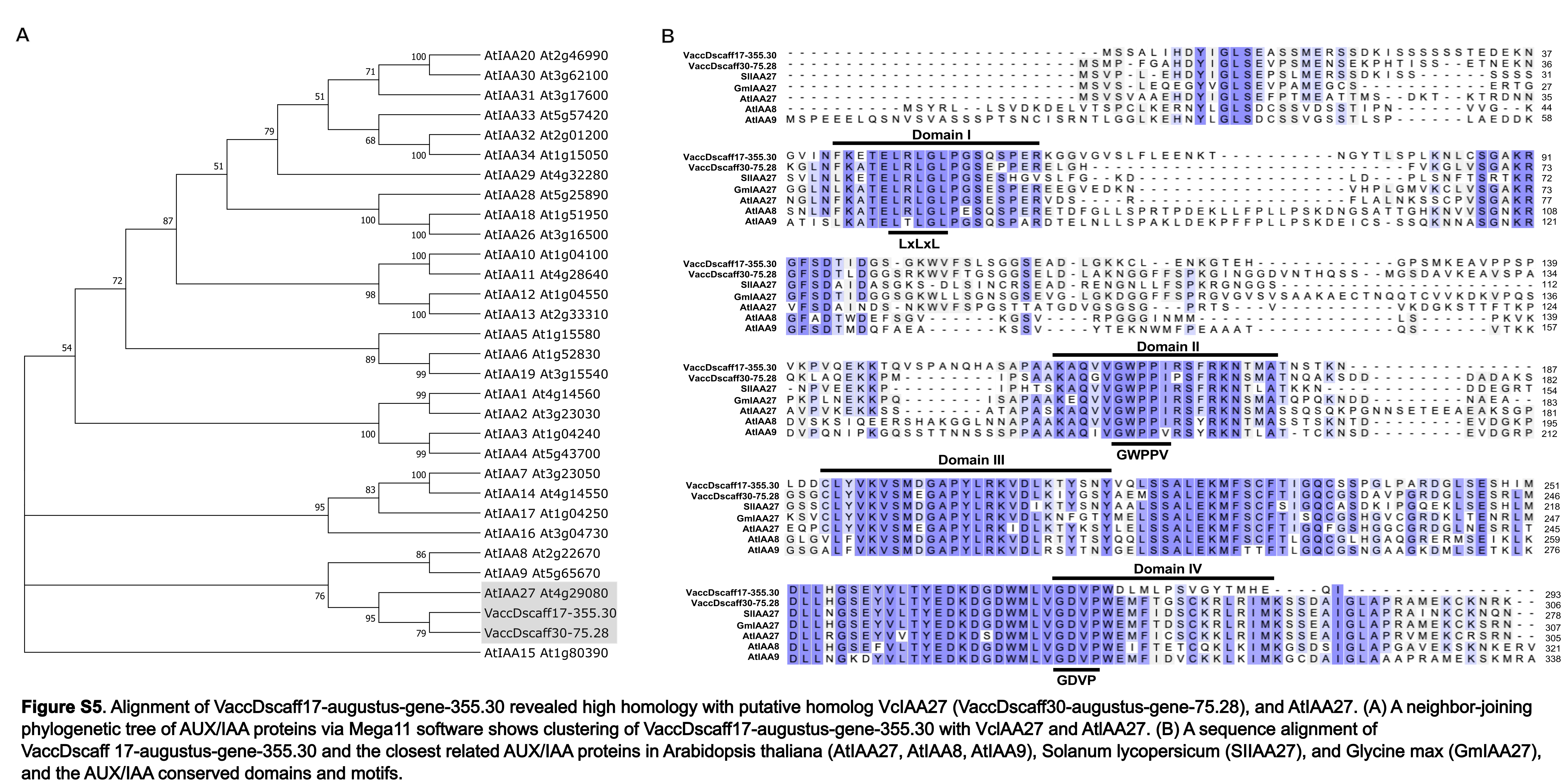

Supplement: Web_Material_uhaf086 [file web_material_uhaf086.zip › S5_AlignmentFigure.png]

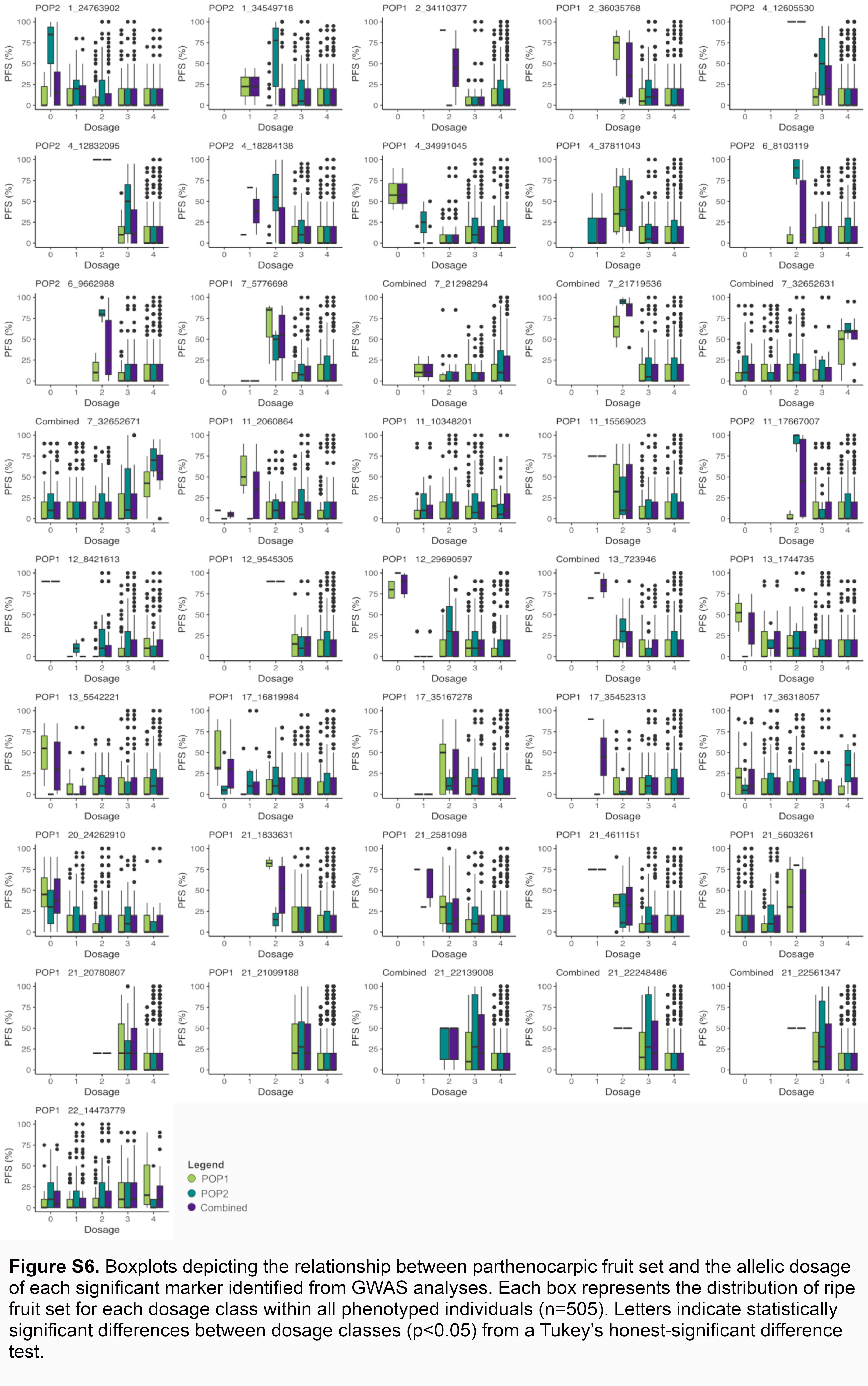

Supplement: Web_Material_uhaf086 [file web_material_uhaf086.zip › S6_MarkerTraitAssociation_N.jpg]

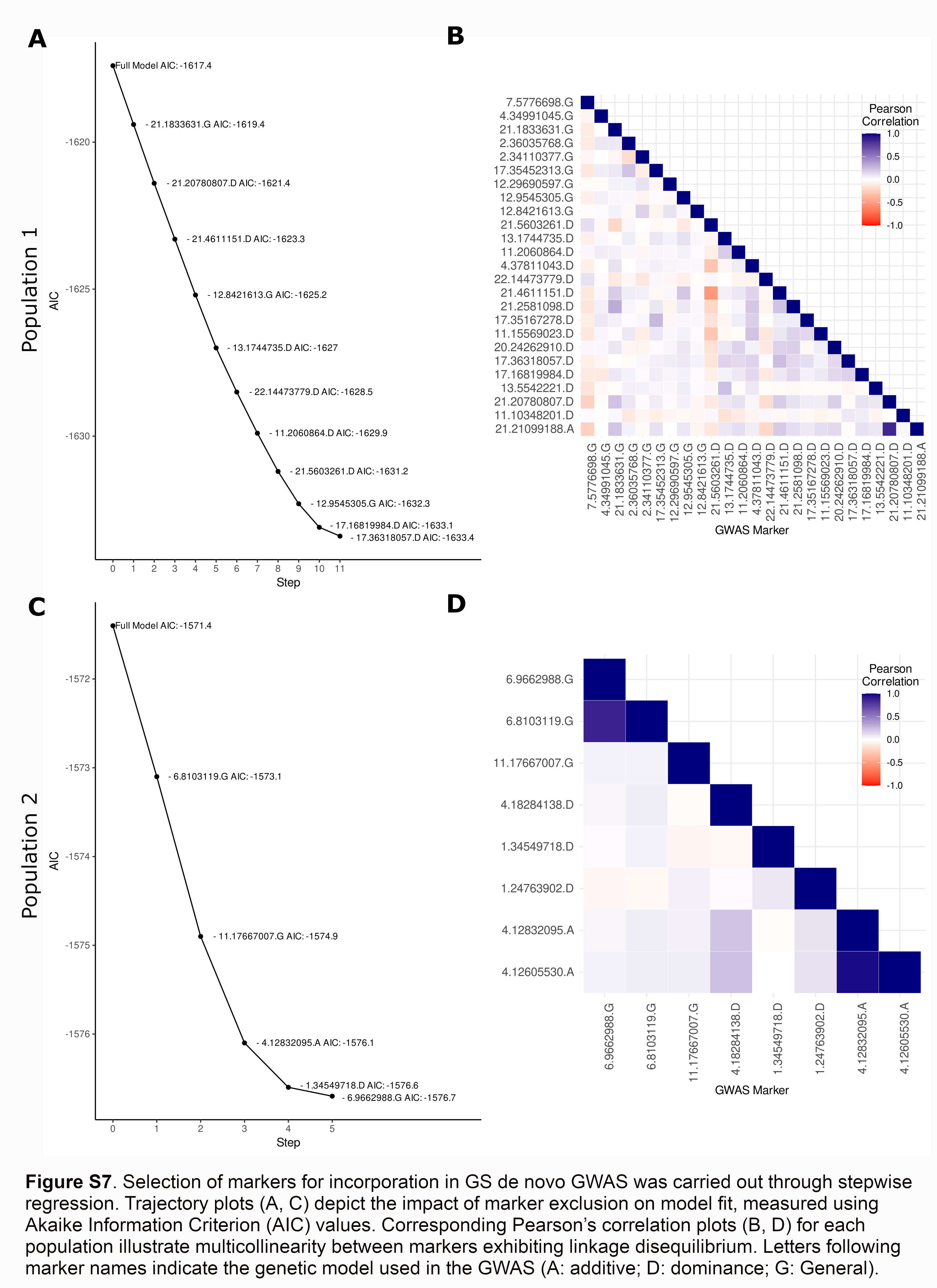

Supplement: Web_Material_uhaf086 [file web_material_uhaf086.zip › S7_StepwiseReg_N.jpg]
